# Comparison of Impairments, Activity Limitations, Balance, and Quality of Life between Patients with and without Meniscus Repair or Partial Meniscectomy Post-ACL Reconstruction

**DOI:** 10.3390/jcm12216933

**Published:** 2023-11-05

**Authors:** Faya Ali Asiri, Abdullah Hassan Assiri, Abdulrhman Abdullh Alqhtani, Mohammed Hassan Alqahtani, Dhuha Saeed Motlag, Jaya Shanker Tedla, Ravi Shankar Reddy, Saad Ali Alwadai

**Affiliations:** 1 Department of Orthopedics, Ahad Rufaidah General Hospital, Abha 62242, Saudi Arabia; drfayaasiri15@gmail.com; 2 Department of Orthopedics, Aseer Central Hospital, Abha 62523, Saudi Arabia; ahf200846@gmail.com (A.H.A.); drdabeel@gmail.com (A.A.A.); altalah2030@gmail.com (M.H.A.); d.motlaq@hotmail.com (D.S.M.); saaalwadai@moh.gov.sa (S.A.A.); 3 Department of Medical Rehabilitation Science, College of Applied Medical Sciences, King Khalid University, Abha 62421, Saudi Arabia; rshankar@kku.edu.sa

**Keywords:** anterior cruciate ligament, reconstruction, meniscus, knee range of motion, knee proprioception error, balance, lower extremity function, quality of life

## Abstract

(1) Background: The anterior cruciate ligament (ACL) is a crucial ligament in the knee joint. This study compares the differences in knee range of motion (ROM), knee proprioception error, balance, function, and quality of life (QOL) among participants with and without meniscus repair or partial meniscectomy nine months post ACL reconstruction. (2) Methods: In this cross-sectional study, 57 male participants were selected through convenience sampling from a tertiary care hospital. Knee flexion and extension ROM were assessed using a digital goniometer; a digital inclinometer was used to assess knee proprioception error; the Y balance test was used to evaluate balance; the lower extremity functional scale (LEFS) was used to assess activity; and QOL was assessed using the ACLQOL questionnaire. (3) Results: There were no significant differences in outcomes except balance. The YB composite score had a moderate negative correlation with knee proprioception error with an R-value of −0.372 **. (4) Conclusions: Nine to 12 months post ACL reconstruction, the isolated ACL reconstruction participants had better lower-quarter single-leg balance than those who underwent ACL reconstruction and meniscal repair or partial meniscectomy. The remaining parameters, like knee ROM, knee proprioception error, LEFS score, and ACLQOL scores, were similar between these two groups.

## 1. Introduction

Injury to the anterior cruciate ligament (ACL) can occur when it becomes stretched or torn [1]. The ACL joins the femur to the tibia and helps stabilize the knee during running, jumping, and pivoting movements [2]. ACL injuries are common among athletes and may happen due to sudden changes in direction, sudden stops, or overextension of the knee [3]. Clinical findings related to ACL injuries include pain, swelling, instability, and a reduced range of motion in the knee. Treatment for an ACL injury may include physical therapy, rest, orthotics, medications, and, in severe cases, surgery [3]. Alzhrani et al. predicted ACL injuries among young athletes in Saudi Arabia. The study found that the incidence rate of ACL injury among young athletes in Saudi Arabia was from 0.1 to 0.4 per 1000 athlete exposures [4,5,6].

There are different types of ACL injuries based on the amount and location of the damage to the ligament. Grade 1 sprains are mild injuries to the ACL where the ligament has been stretched but not torn. Grade 2 sprains are moderate injuries where the ACL is partially torn but still has some functional ability. Grade 3 sprains are severe injuries resulting in a complete ACL rupture. Furthermore, there are two types of ACL tears based on the location of the tear. Mid-substance tears occur in the middle of the ligament and are the most common type of ACL injury, while avulsion tears occur when the ligament is torn off the bone to which it is attached, often taking a small piece of bone with it [7,8].

In an isolated ACL injury, surgical management typically involves reconstructing the ACL using a graft from the patient’s or a donor’s tissue. Arthroscopic surgery is the most common surgical approach, which is less invasive than open surgery and involves making small incisions in the knee joint. The postoperative rehabilitation program typically includes controlled ROM exercises, muscle strengthening, and sports-specific training. Patients typically return to sports activities about six to nine months after surgery [8,9].

In an ACL injury where the meniscus is damaged, surgical management depends on the severity of the meniscus injury. It can involve either meniscus repair or meniscectomy (partial or complete removal of the damaged meniscus). If a meniscus repair is performed, the rehabilitation program will be more conservative and involve protected weight-bearing for longer to allow the meniscus to heal. If a meniscectomy is performed, the rehabilitation program will focus on reconstructing the ACL and follow a similar program as an isolated ACL injury. However, patients with a meniscectomy may experience a higher incidence of early-onset osteoarthritis than those with an intact meniscus. Managing an ACL with a meniscus injury is complex and requires careful consideration of the individual patient’s injury [10,11].

An ACL injury can result in impairments like muscle weakness, restricted ROM, and proprioception deficits [12]. Muscle weakness or atrophy of the quadriceps and hamstring muscles has been reported in individuals with ACL injuries [13]. Graft harvesting during reconstruction surgery can also lead to further muscle weakness. Proprioception is the capacity to sense joint position and movement; an ACL injury can impair proprioception, leading to difficulty with precise joint movements. Impaired proprioception can cause balance and gait deficits. These impairments can interfere with function recovery following an ACL injury. Rehabilitation programs aim to restore muscle strength, ROM, and proprioception and usually consist of progressive exercises over 12 months [14].

After an ACL injury, activity limitations may cause difficulty maintaining stability when standing on one leg or standing still. These difficulties can result in unsteadiness and instability that may cause limping, an altered gait pattern, or difficulty walking on uneven surfaces or stairs [15,16,17].

Limited research has been conducted on improvements in these impairments and activity limitations among patients with ACL injuries with and without meniscal damage. Therefore, this study aims to determine the differences between two types of surgeries, ACL reconstruction alone versus ACL reconstruction with meniscus repair or partial meniscectomy, and evaluate their impacts on impairments and functional limitations.

## 2. Materials and Methods

This study obtained certificated ethical clearance with approval number ECM#2023-302 from King Khalid University’s research ethics committee (HAPO-06-B-001). In total, 57 participants were recruited from a hospital or clinic setting. The website https://clincalc.com/Stats/SampleSize.aspx accessed on 25 September 2023 was used to estimate sample size. The design used in this website for the sample size calculation was one research group versus the population. The expected research group mean was determined based on the known mean from previous literature. The power of the study was kept at 80%, and the chance of type I error, or alpha, was set at 0.05. The researchers calculated that 52 participants would be adequate; however, assuming a 10% dropout rate, the sample size was 57. The study lasted a year and was carried out at King Khalid University in Abha.

The participants had either undergone ACL reconstruction alone, or ACL reconstruction with meniscus repair or partial meniscectomy. The inclusion criteria were as follows: participants should be between 18 and 60 years old, have a history of ACL injury and surgical reconstruction, and be between nine and 12 months post surgery. Patients with other knee-related problems, surgeries, and other medical conditions that may affect knee function were excluded from the study.

### 2.1. Knee ROM Assessment

**Figure 1:**
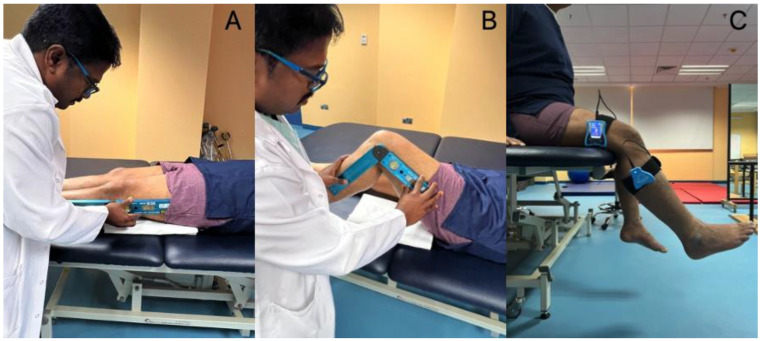
Demonstrating the outcome measurement procedures. (**A**) Measuring knee extension range of motion; (**B**) measuring knee flexion range of motion; (**C**) measuring knee proprioception using the inclinometer.

A Baseline^®^ Absolute+Axis^®^ Digital Goniometer, Fabrication Enterprises, Elmsford, NY, USA measured ROM for knee flexion and extension. The participants were put in a comfortable supine position. They were given a 2 cm thick rubber cushion to place beneath their thighs, allowing their knee extension to be evaluated more easily. The therapist placed the lateral knee aspect with a digital goniometer with a precision of 0.1°. The goniometer touched the patient’s skin, and the stable arm was parallel to the thigh. The fulcrum was at the knee joint line, and the movable arm was along the midline of the leg. The therapist held the goniometer firmly, as shown in Figure 1. The participants were asked to bend and flex their knees actively, and the angles of their full range were recorded in degrees. Each ROM measurement was performed three times, and an average of the three measurements was considered for final analysis [18,19].

### 2.2. Knee Proprioception Measurement

The participants were asked to sit up straight in the chair. The researchers used a Dualer IQ Digital Inclinometer to evaluate knee proprioception in the open kinetic chain position. Using a strap, the researchers attached a portion of the inclinometer to the lower third of the femur’s lateral aspect. The second portion of the inclinometer was fastened to the upper third of the leg on the lateral aspect (Figure 1). From the beginning position of knee flexion at 90 degrees, participants were asked to extend their knees until they reached the desired angle of 30°. The researchers showed the participants the 30° goal angle three times before asking them to replicate it three more times. The angles that deviated from the exact 30-degree position were recorded as knee proprioception error angles [20].

### 2.3. Y-Balance Test for the Lower Quarter

The Y-balance tool kit was placed on the floor. The standard testing protocol prescribed in the textbooks was followed. The participants stood with their affected legs on a stance plate with their toes touching the red line. The researchers asked them to move their other leg anteriorly and then move the marker on the pipe forward without completely placing their foot on the floor to its maximum capacity. The leg should move in posterior lateral and posterior medial directions. Three trials were performed in each direction, and the average distance covered was recorded as their performance distance [21,22].

### 2.4. ACLQOL Questionnaire

The ACLQOL questionnaire evaluates problems and clinical features, occupational concerns, involvement in sports and leisure activities, lifestyle, community participation, and psychological health concerning knee and anterior cruciate ligament injuries. It is a subjective, patient-based evaluation tool. The 31 items on this instrument are created to evaluate patients’ quality of life (QOL) with chronic ACL injuries. They are divided into five categories: symptoms and physical complaints, work-related concerns, participation in leisure activities and competitive sports, lifestyle, and social and emotional aspects. The researchers used the English version of the ACLQOL during an interview with each participant [23,24,25,26].

### 2.5. LEFS

The LEFS is a questionnaire with 20 questions. It is a self-reported test that assesses the functional posture of patients with musculoskeletal-based disorders of the lower limbs. It assesses patients on a Likert scale with five points. A score of 80 denotes an individual with normal function, and a score of zero indicates extreme difficulty or inability to perform each of the mentioned activities. This method is frequently used to determine functional capacity following ACL reconstructions [27,28,29].

### 2.6. Data Analysis

The Statistical Package for Social Sciences, version 24, was used to analyze the variables. The significance level was kept at *p* values < 0.05 with a confidence interval of 95%. The mean, SD, minimum, and maximum values of demographic characteristics and outcome-related information for all the participants were descriptively analyzed. The differences in variables between the two types of injuries and the two types of surgeries were evaluated using the Mann–Whitney U test. The correlations between various study parameters were performed with Spearman rank correlation tests.

## 3. Results

This study aimed to identify differences in ROM, proprioception, lower extremity function, balance, and QOL between patients who had undergone ACL reconstruction alone versus ACL reconstruction with meniscal repair or partial meniscectomy. All participants were men, and the total number of participants recruited for the study was 57. The participants’ mean ± SD age was 30.64 ± 6.24. Among them, 31 had isolated ACL injuries, and 26 had ACL injuries with meniscus injuries. Table 1 provides details of their demographic characteristics in terms of mean, SD, minimum, and maximum.

Patient-related factors like age, height, lower limb length, weight, and body mass index (BMI) were compared between the patients in the ACL injury alone and the ACL with meniscus injury groups. Weight and BMI were higher in the ACL with meniscal injury group than in the isolated ACL injury group, with a statistically significant difference of *p* < 0.05. Patient-related factors like knee flexion range of motion, knee proprioception error, YBT direction reach distances, ACLQOL questionnaire score, LEFS score, and YBT cumulative and composite scores were compared between the groups. YBT posterolateral direction reach distances and YBT composite scores were higher in the isolated ACL injury group than in the ACL with meniscal injury group, with a statistically significant difference of *p* < 0.05. The knee extension range of motion was full (zero lag) in all the participants, so the knee extension scores are not presented in the tables. Table 2 provides additional details for these parameters, including their means, SD, and *p* values.

The above-mentioned patient-related factors were also compared between the two types of surgeries performed: ACL reconstruction alone or ACL reconstruction with meniscal repair or partial meniscectomy. YBT posterolateral direction reach distances and YBT composite scores were higher in isolated ACL reconstruction than in the ACL reconstruction with the meniscal repair group, with a statistically significant difference of *p* < 0.05. Table 3 provides details of these parameters, including their means, SD, and *p* values. Correlation analyses were performed between demographic and patient-related characteristics; their results are presented in Table 4.

There was an excellent correlation between height and LLL (0.935 **), BMI and weight (0.865 **), and YB cumulative and composite scores (0.846 **). A good correlation was found between the type of surgery and the type of injury (0.781 **). There was a moderate correlation between LEFS and ACLQOL (0.553 **), ACLQOL and BMI (0.478 **), ACLQOL and weight (0.344 **), weight and LLL (0.347**), type of injury and BMI (0.344 **), and type of injury and weight (0.409 **). A minimum correlation was found between weight and age (0.295 *), weight and height (0.295 *), knee proprioception and side (0.286 *), and LEFS and BMI (0.286 *). There was a moderate negative correlation between ACLQOL and duration (−0.394 **), LEFS and age (−0.3304 *), YB cumulative score and duration (−0.364 **), YB cumulative score and knee proprioception (−0.388 **), YB composite score and LLL (−0.353 **), YB composite score and weight (−0.354 **), YB composite score and type of injury (−0.326 **), YB composite score and duration of injury (−0.326 *), and YB composite score and knee proprioception (−0.372 **). There was a minimal negative correlation between LEFS and height (−0.274 *) and YB composite score and height (−0.288 *).

## 4. Discussion

This unique cross-sectional study has compared critical clinical outcomes like knee flexion ROM, knee proprioception error, balance, LEFS score, and QOL parameters between patients with isolated ACL reconstruction and patients with ACL reconstruction with meniscal repair or partial meniscectomy nine months post surgery. The study has identified some notable findings. For example, these parameters were the same between the two types of surgery except the balance scores. The total Y balance composite scores were better in isolated ACL reconstruction patients than in ACL with meniscus repair or partial meniscectomy patients, with a *p* value less than 0.05.

Pua et al. [30] conducted a long-term study on patients with ACL reconstructions and assessed their functional parameters up to seven months post surgery. In their study, the average knee flexion ranges were more than 130 degrees, reflecting this study’s findings. A meta-analysis conducted by Relph et al. on knee proprioception post ACL injury compared knee proprioception errors between the affected and non-affected legs using dynamometers and purposefully made proprioceptive measuring devices. They found the error was up to seven degrees in the affected knee. This study calculated an average error of 13 degrees, which is high compared with other studies [14]. These differences may be because this study used inclinometers, while Relph et al. used dynamometers.

The Y balance test scores of affected ACL limbs observed in this study were comparable to the findings of Oleksy et al. [31]. This study’s anterior, posterolateral, posteromedial, and composite scores were 67.52, 96.08, 92.18, and 92.98 cm, respectively. In Oleksy et al.’s study, they were 69.2, 103.2, 109.2, and 93.9, respectively [31]. In this study, measurements were conducted 9 months post surgery, whereas in Oleksy et al.’s study, measurements were taken 24 months post surgery; this explains why their values were higher than those in this study.

Grant et al. [32] followed post-ACL reconstruction patients for two to four years and compared improvements in QOL between home-based program participants and physical therapy-supervised participants. They found ACLQOL scores ranging from 69.9 to 80 out of 100. This study found an average score of 73.31 out of 100. Grant et al.’s values were slightly higher [32]. These higher values may be due to the duration of follow-up, which was two to four years, whereas in this study, it was nine to twelve months.

Greg et al. [33] observed long-term changes in LEFS scores up to 16 weeks, around four months, post-ACL reconstruction. The participants reached a maximum score of 63 out of 80. This study assessed the LEFS after nine months. This study observed an average score of 71.77, higher than Greg et al.’s study. This may be due to the differences in the assessment duration of all the parameters; only balance, measured by lower quarter Y balance test composite scores, was significantly higher in isolated ACL reconstruction participants than in ACL reconstruction with meniscal repair or partial meniscectomy participants. These differences may be due to a lack of proper feedback from knee joint structures like the meniscus during this dynamic, single-leg, close kinematic balance activity. This hypothesis was also observed in Parus et al.’s study. They observed abnormalities in body balance control among participants who had undergone ACL reconstruction with medial meniscal sutures [34]. Another cadaveric study by Shoemaker and Markolf demonstrated the importance of the meniscus in the anterior–posterior stability of a knee joint without an ACL [35]. They highlight that the lateral meniscectomy, followed by the medial meniscectomy, resulted in a further 10% rise in overall anterior–posterior mobility assessed at 200 newtons of applied tibial force. When a medial meniscus rupture with a bucket handle was repaired, the tibia subluxated anteriorly on the femur due to the joint force, altering the knee’s stability. These biomechanical observations and a deficit in close kinetic proprioception could have contributed to the balance differences.

### Limitations

As this was a cross-sectional study, the researchers did not analyze the patients’ experiences before and after the evaluation period. The study only included male samples due to cultural restrictions in the study region. Furthermore, ACL injuries and reconstructions were rare in women in this region. The values of the affected people were neither compared with a normative group nor their normal side. This comparison in the future could reveal some interesting improvements. Furthermore, this study focused on patients undergoing surgery at a single center. Therefore, a prospective, multicentric trial including female participants could improve the study findings. Moreover, future studies should be performed comparing the outcomes of isolated groups incorporating normal subjects, subjects with ACL injury without surgery, subjects with only ACL reconstruction, subjects with ACL reconstruction with meniscal repair, and subjects with ACL reconstruction with meniscal removal. These comparisons will provide a much clearer understanding of outcomes and improvements among ACL-injured patients.

## 5. Conclusions

Balance was the only significant difference between pure ACL reconstructions and ACL reconstructions with meniscal repair or partial meniscectomy. There were no significant differences between the groups related to any remaining parameters, like knee ROM, knee proprioception, lower extremity function, and QOL, nine months post ACL reconstruction.

## Figures and Tables

**Table 1:** Demographic characteristics and outcome-related information of all the subjects.

Variables	Mean	SD	Minimum	Maximum
Age	30.47	6.24	18.00	52.00
Height	1.70	0.05	1.63	1.80
Lower limb length	91.82	4.38	80.00	100.00
Weight	76.16	7.99	55.00	92.00
Body Mass Index	26.18	2.73	19.84	31.93
Duration (Months)	16.25	14.49	4.00	108.00
Knee flexion range of motion	124.76	8.91	101.67	140.00
Knee Proprioception (45°)	13.81	3.56	5.00	23.33
Lower Extremity Functional Scale score (out of 80)	71.77	8.39	39.00	80.00
YBT anterior direction reach distance	67.52	9.28	43.67	94.00
YBT posteromedial direction reach distance	92.18	9.05	71.67	110.33
YBT posterolateral direction reach distance	96.08	10.50	65.00	120.33
YBT three direction reach distance cumulative score	255.78	21.80	204.00	304.33
YBT composite score	92.98	8.13	71.89	108.17
ACL Quality of Life Questionnaire Score (out of 100)	73.31	17.90	27.81	97.81

Note—YBT: Y balance test; ACL: anterior cruciate ligament; SD: standard deviation.

**Table 2:** Comparison of variables between the two types of injuries.

Variables	ACL Isolated Injury (31) (Mean ± SD)	ACL with Meniscus Injury (26) (Mean ± SD)	*p* Value	Cohen’s d	SE Cohen’s d
Age (years)	29.03 ± 5.70	32.19 ± 6.52	0.056	−0.519	0.274
Height (meters)	1.70 ± 0.05	1.71 ± 0.05	0.427	−0.213	0.267
Lower Limb Length (cm)	91.16 ± 4.52	92.62 ± 4.16	0.215	−0.334	0.269
Weight (kilograms)	73.19 ± 8.91	79.69 ± 4.87	0.002 **	−0.883	0.289
Body Mass Index (kg/m^2^)	25.32 ± 3.03	27.19 ± 1.91	0.009 **	−0.722	0.281
Knee Flexion ROM (degrees)	122.97 ± 9.11	126.88 ± 8.34	0.099	−0.447	0.272
Knee Proprioception Error (degrees)	13.18 ± 2.95	14.56 ± 4.10	0.146	−0.392	0.271
YBT Anterior DRD (cm)	67.94 ± 9.86	67.02 ± 8.68	0.713	0.098	0.266
YBT Posteromedial DRD (cm)	92.95 ± 9.34	91.26 ± 8.78	0.487	0.186	0.267
YBT Posterolateral DRD (cm)	99.72 ± 8.55	91.74 ± 11.09	0.003 **	0.815	0.285
ACL QOL Questionnaire Score (100)	70.44 ± 20.10	76.74 ± 14.50	0.189	−0.354	0.270
LEFS score (80)	70.97 ± 10.05	72.73 ± 5.88	0.434	−0.209	0.267
YBT Cummilative score	260.61 ± 18.33	250.02 ± 24.45	0.067	0.496	0.273
YBT Composite score	95.39 ± 6.43	90.11 ± 9.09	0.013 *	0.681	0.280

Note—YBT: Y balance test; ACL: anterior cruciate ligament; SD: standard deviation; cm: centimeter; kg: kilogram; m: meter; DRD: direction reach distance; QOL: quality of life; LEFS: lower extremity functional score. * Indicates level of significance *p*-value is less than 0.05. ** Indicates level of significance *p*-value is less than 0.01.

**Table 3:** Comparison of patient-related variables and study outcomes between the two types of surgeries.

Variables	ACLR Alone (24) (Mean ± SD)	ACLR with Meniscal Repair or Removal (33) (Mean ± SD)	*p* Value	Cohen’s d	SE Cohen’s d
Age (years)	28.71 ± 6.16	31.76 ± 6.06	0.07	−0.500	0.278
Height (meters)	1.69 ± 0.04	1.71 ± 0.05	0.06	−0.526	0.279
Lower Limb Length (cm)	90.54 ± 3.34	92.76 ± 4.84	0.06	−0.518	0.279
Weight (kilograms)	74.46 ± 8.21	77.39 ± 7.71	0.17	−0.370	0.274
Body Mass Index (kg/m^2^)	26.03 ± 2.78	26.28 ± 2.73	0.74	−0.090	0.269
Knee Flexion ROM (degrees)	123.14 ± 7.84	125.93 ± 9.55	0.25	−0.314	0.272
Knee Proprioception Error (degrees)	13.57 ± 2.93	13.99 ± 3.99	0.66	−0.117	0.269
YBT Anterior DRD (cm)	67.37 ± 6.34	67.63 ± 11.04	0.91	−0.028	0.268
YBT Posteromedial DRD (cm)	93.06 ± 10.13	91.54 ± 8.28	0.54	0.167	0.269
YBT Posterolateral DRD (cm)	100.24 ± 7.37	93.06 ± 11.46	0.01	0.721	0.288
ACL QOL Questionnaire Score (100)	73.11 ± 20.87	73.46 ± 15.74	0.94	−0.020	0.268
LEFS score (80)	73.08 ± 9.00	70.82 ± 7.92	0.32	0.270	0.271
YBT Cummilative score	260.66 ± 17.44	252.23 ± 24.12	0.15	0.391	0.274
YBT Composite score	95.97 ± 5.55	90.81 ± 90.05	0.01	0.663	0.285

Note—YBT: Y balance test; ACL: anterior cruciate ligament; SD: standard deviation; cm: centimeter; kg: kilogram; m: meter; DRD: direction reach distance; QOL: quality of life; LEFS: lower extremity functional score.

**Table 4:** Correlation between the demographic characteristics and outcome measures.

Variables	KFROMM	KPTM	ACLQOL (100)	LEFS (80)	YB Cumulative Score	YB Composite Score
Age	−0.21	0.078	−0.146	−0.304 *	−0.14	−0.245
Height	−0.084	−0.015	−0.196	−0.274 *	0.221	−0.288 **
LLL	0.04	0.013	−0.081	−0.135	0.198	−0.353 **
Weight	−0.199	0.172	0.344 **	0.15	−0.172	−0.354 **
BMI	−0.168	0.182	0.478 **	0.286 *	−0.247	−0.174
Side	−0.114	0.286 *	0.105	0.032	0.034	−0.048
Duration (M)	0.197	−0.09	−0.394 **	−0.147	−0.364 *	−0.330 *
T Injury	0.221	0.195	0.177	0.106	−0.244	−0.326 *
T Surgery	0.156	0.059	0.01	−0.135	−0.193	−0.316 *
KFROMM	1	−0.239	0.078	0.17	0.115	0.069
KPTM	−0.239	1	0.15	−0.02	−0.388 **	−0.372 *
ACLQOL (100)	0.078	0.15	1	0.553 **	−0.107	−0.065
LEFS (80)	0.17	−0.02	0.553 **	1	0.136	0.196
YB Cumulative score	0.115	−0.388 **	−0.107	0.136	1	0.846 **

* Correlation is significant at the 0.05 level (2-tailed); ** Correlation is significant at the 0.01 level (2-tailed). LLL: Lower Limb Length, M: Months, T: Type of, KFROMM: Knee Flexion Range Of Motion Mean, KPTM: Knee Proprioception Test Mean, ACLQOL: anterior cruciate ligament quality of life questionnaire, LEFS: lower extremity functional score, YB: Y balanceTest.

## Data Availability

Data are available with the corresponding author mentioned in this research paper.

## References

[1] Årøen A., Sivertsen E.A., Owesen C., Engebretsen L., Granan L.P. (2013). An isolated rupture of the posterior cruciate ligament results in reduced preoperative knee function in comparison with an anterior cruciate ligament injury. Knee Surg. Sports Traumatol. Arthrosc..

[2] Gupton M., Imonugo O., Terreberry R.R. (2018). Anatomy, Bony Pelvis and Lower Limb, Knee.

[3] Vilks S. (2021). Knee Anterior Cruciate Ligament Injury in Sports. Lase J. Sport Sci..

[4] Alenezi F.S. (2016). The Relationship between Lower Limb Biomechanical Variables during Common Screening Tasks.

[5] Alzhrani M.M. (2018). Biomechanical Measures of Lower Limb Variability, and Prediction of Non-Contact Knee Injuries Risk Factors in Male Athletes.

[6] Prieto-González P., Martínez-Castillo J.L., Fernández-Galván L.M., Casado A., Soporki S., Sánchez-Infante J. (2021). Epidemiology of sports-related injuries and associated risk factors in adolescent athletes: An injury surveillance. Int. J. Environ. Res. Public Health.

[7] McAllister D.R., Parker R.D., Cooper A.E., Recht M.P., Abate J. (1999). Outcomes of postoperative septic arthritis after anterior cruciate ligament reconstruction. Am. J. Sports Med..

[8] Van Melick N., Van Cingel R.E.H., Brooijmans F., Neeter C., van Tienen T., Hullegie W., Nijhuis-van der Sanden M.W.G. (2016). Evidence-based clinical practice update: Practice guidelines for anterior cruciate ligament rehabilitation based on a systematic review and multidisciplinary consensus. Br. J. Sports Med..

[9] Fabricant P.D., Kocher M.S. (2017). Management of ACL injuries in children and adolescents. J. Bone Jt. Surg..

[10] Abram S.G.F., Judge A., Beard D.J., Price A.J. (2019). Rates of adverse outcomes and revision surgery after anterior cruciate ligament reconstruction: A study of 104,255 procedures using the National Hospital Episode Statistics Database for England, UK. Am. J. Sports Med..

[11] Logan C.A., Aman Z.S., Kemler B.R., Storaci H.W., Dornan G.J., LaPrade R.F. (2019). Influence of medial meniscus bucket-handle repair in setting of anterior cruciate ligament reconstruction on tibiofemoral contact mechanics: A biomechanical study. Arthrosc. J. Arthrosc. Relat. Surg..

[12] Grindem H., Eitzen I., Moksnes H., Snyder-Mackler L., Risberg M.A. (2012). A pair-matched comparison of return to pivoting sports at 1 year in anterior cruciate ligament–injured patients after a nonoperative versus an operative treatment course. Am. J. Sports Med..

[13] Relph N., Herrington L., Tyson S. (2014). The effects of ACL injury on knee proprioception: A meta-analysis. Physiotherapy.

[14] Shelbourne K.D., Gray T., Haro M. (2009). Incidence of subsequent injury to either knee within 5 years after anterior cruciate ligament reconstruction with patellar tendon autograft. Am. J. Sports Med..

[15] Risberg M.A., Holm I., Myklebust G., Engebretsen L. (2007). Neuromuscular training versus strength training during first 6 months after anterior cruciate ligament reconstruction: A randomized clinical trial. Phys. Ther..

[16] Frank B.S., Gilsdorf C.M., Goerger B.M., Prentice W.E., Padua D.A. (2014). Neuromuscular fatigue alters postural control and sagittal plane hip biomechanics in active females with anterior cruciate ligament reconstruction. Sports Health.

[17] Patterson B.E., Barton C.J., Culvenor A.G., Cooper R.L., Crossley K.M. (2021). Exercise-therapy and education for individuals one year after anterior cruciate ligament reconstruction: A pilot randomised controlled trial. BMC Musculoskelet. Disord..

[18] Clarkson H.M. (2020). Musculoskeletal Assessment: Joint Range of Motion, Muscle Testing, and Function.

[19] Reese N.B., Bandy W.D., Yates C. (2010). Measurement of range of motion of the knee. Joint Range of Motion and Muscle Length Testing.

[20] Suner-Keklik S., Cobanoglu-Seven G., Kafa N., Ugurlu M., Guzel N.A. (2017). The validity and reliability of knee proprioception measurement performed with inclinometer in different positions. J. Sport Rehabil..

[21] Chakrabarti D., Karmakar S., Salve U.R. (2022). Ergonomics for Design and Innovation: Humanizing Work and Work Environment: Proceedings of HWWE 2021.

[22] Kotwal P.P., Mittal K. (2020). Joshi and Kotwal’s Essentials of Orthopedics and Applied Physiotherapy-E-Book.

[23] Mohtadi N. (1998). Development and validation of the quality of life outcome measure (questionnaire) for chronic anterior cruciate ligament deficiency. Am. J. Sports Med..

[24] Silva L.O., Mendes L.M.R., de Paula Lima P.O., Almeida G.P.L. (2018). Translation, cross-adaptation and measurement properties of the Brazilian version of the ACL-RSI Scale and ACL-QoL Questionnaire in patients with anterior cruciate ligament reconstruction. Braz. J. Phys. Ther..

[25] Filbay S.R., Culvenor A.G., Ackerman I.N., Russell T.G., Crossley K.M. (2015). Quality of life in anterior cruciate ligament-deficient individuals: A systematic review and meta-analysis. Br. J. Sports Med..

[26] Kinikli G.I., Celik D., Yuksel I., Atay O.A. (2015). Turkish version of the anterior cruciate ligament quality of life questionnaire. Knee Surg. Sports Traumatol. Arthrosc..

[27] Korakakis V., Saretsky M., Whiteley R., Azzopardi M.C., Klauznicer J., Itani A., Al Sayrafi O., Giakas G., Malliaropoulos N. (2019). Translation into modern standard Arabic, cross-cultural adaptation and psychometric properties’ evaluation of the Lower Extremity Functional Scale (LEFS) in Arabic-speaking athletes with Anterior Cruciate Ligament (ACL) injury. PLoS ONE.

[28] Mehta S.P., Fulton A., Quach C., Thistle M., Toledo C., Evans N.A. (2016). Measurement properties of the lower extremity functional scale: A systematic review. J. Orthop. Sports Phys. Ther..

[29] Alnahdi A.H., Alrashid G.I., Alkhaldi H.A., Aldali A.Z. (2016). Cross-cultural adaptation, validity and reliability of the Arabic version of the Lower Extremity Functional Scale. Disabil. Rehabil..

[30] Pua Y.-H., Low J., Woon E.-L., Tay O.S.-M., Cheong P., Thumboo J., Clark R.A., Chang P., Tan A., Ho J.-Y. (2021). Knee performance and self-efficacy trajectory curves after ACL reconstruction: A longitudinal study. Phys. Ther. Sport.

[31] Oleksy Ł., Mika A., Sulowska-Daszyk I., Szymczyk D., Kuchciak M., Stolarczyk A., Rojek R., Kielnar R. (2021). Standard RTS criteria effectiveness verification using FMS, Y-balance and TJA in footballers following ACL reconstruction and mild lower limb injuries. Sci. Rep..

[32] Grant J.A., Mohtadi N.G.H. (2010). Two-to 4-year follow-up to a comparison of home versus physical therapy-supervised rehabilitation programs after anterior cruciate ligament reconstruction. Am. J. Sports Med..

[33] Alcock G.K., Werstine M.S., Robbins S.M., Stratford P.W. (2012). Longitudinal changes in the lower extremity functional scale after anterior cruciate ligament reconstructive surgery. Clin. J. Sport Med..

[34] Parus K., Lisiński P., Huber J. (2015). Body balance control deficiencies following ACL reconstruction combined with medial meniscus suture. A preliminary report. Orthop. Traumatol. Surg. Res..

[35] Shoemaker S.C., Markolf K.L. (1986). The role of the meniscus in the anterior-posterior stability of the loaded anterior cruciate-deficient knee. Effects of partial versus total excision. J. Bone Jt. Surg..

